# Practical Guidelines by the Andalusian Group for Nutrition Reflection and Investigation (GARIN) on Nutritional Management of Patients with Chronic Obstructive Pulmonary Disease: A Review

**DOI:** 10.3390/nu16183105

**Published:** 2024-09-14

**Authors:** Alicia Justel Enríquez, Juana M. Rabat-Restrepo, Francisco J. Vilchez-López, Carmen Tenorio-Jiménez, José M. García-Almeida, José-Antonio Irles Rocamora, José L. Pereira-Cunill, María J. Martínez Ramírez, María J. Molina-Puerta, Juan B. Molina Soria, María I. Rebollo-Pérez, Gabriel Olveira, Pedro P. García-Luna

**Affiliations:** 1 Servicio de Endocrinología y Nutrición, Hospital Universitario de la Princesa, 28006 Madrid, Spain; 2 Servicio de Endocrinología y Nutrición, Hospital Universitario Virgen Macarena, 41009 Sevilla, Spain; 3 Departamento de Medicina, Facultad de Medicina, Universidad de Sevilla, 41009 Sevilla, Spain; irles@us.es (J.-A.I.R.); garcialunapp@yahoo.es (P.P.G.-L.); 4 Servicio de Endocrinología y Nutrición, Hospital Puerta del Mar, 11009 Cádiz, Spain; 5 Endocrinology and Nutrition Clinical Management Unit, University Hospital Virgen de las Nieves, 18014 Granada, Spain; 6 Unidad de Gestión Clínica de Endocrinología y Nutrición, Hospital Universitario Virgen de la Victoria, 29010 Málaga, Spain; 7 Instituto de Investigación Biomédica de Málaga/Plataforma Bionand, 29010 Málaga, Spain; 8 UGC Endocrinología y Nutrición, Hospital Universitario Valme, 41014 Sevilla, Spain; 9 Unidad de Gestión Clínica de Endocrinología y Nutrición, Hospital Universitario Virgen del Rocío, 41013 Sevilla, Spain; 10 Endocrine Diseases Research Group, Institute of Biomedicine of Seville (IBIS), 41007 Sevilla, Spain; 11 Servicio de Endocrinología y Nutrición, Complejo Hospitalario de Jaén, 23007 Jaén, Spain; 12 Facultad de Medicina, Universidad de Jaén, 23071 Jaén, Spain; 13 UGC Endocrinología y Nutrición, Hospital Universitario Reina Sofía, 14004 Córdoba, Spain; 14 Instituto Maimónides de Investigación Biomédica de Córdoba (IMIBIC), 14004 Córdoba, Spain; 15 Unidad de Nutrición y Dietética, Hospital General, 23700 Linares, Spain; 16 Servicio de Endocrinología y Nutrición, Hospital Juan Ramón Jiménez, 21005 Huelva, Spain; 17 Unidad de Gestión Clínica de Endocrinología y Nutrición, Hospital Regional Universitario de Málaga, 29010 Málaga, Spain; 18 Centro de Investigación Biomédica en Red de Diabetes y Enfermedades Metabólicas Asociadas (CIBERDEM), Instituto de Salud Carlos III, 28029 Madrid, Spain; 19 Departamento de Medicina y Dermatología, Facultad de Medicina, Universidad de Málaga, 29010 Málaga, Spain

**Keywords:** chronic obstructive pulmonary disease, nutrition, diet, enteral nutrition

## Abstract

Malnutrition is common in chronic obstructive pulmonary disease (COPD) patients and is associated with worse lung function and greater severity. This review by the Andalusian Group for Nutrition Reflection and Investigation (GARIN) addresses the nutritional management of adult COPD patients, focusing on Morphofunctional Nutritional Assessment and intervention in clinical practice. A systematic literature search was performed using the Preferred Reporting Items for Systematic Reviews and Meta-Analyses (PRISMA) methodology, followed by critical appraisal based on Scottish Intercollegiate Guidelines Network (SIGN) guidelines. Recommendations were graded according to the European Society for Clinical Nutrition and Metabolism (ESPEN) system. The results were discussed among GARIN members, with consensus determined using a Likert scale. A total of 24 recommendations were made: 2(A), 6(B), 2(O), and 14(GPP). Consensus exceeded 90% for 17 recommendations and was 75–90% for 7. The care of COPD patients is approached from a nutritional perspective, emphasizing nutritional screening, morphofunctional assessment, and food intake in early disease stages. Nutritional interventions include dietary advice, recommendations on food group intake, and the impact of specialized nutritional treatment, particularly oral nutritional supplements. Other critical aspects, such as physical activity and quality of life, are also analyzed. These recommendations provide practical guidance for managing COPD patients nutritionally in clinical practice.

## 1. Introduction

Chronic obstructive pulmonary disease (COPD) is the third leading cause of death worldwide, amounting to 3 million in 2016 [1]. It is also associated with morbidity and disability [2,3] and usually presents with other associated comorbidities, including malnutrition [4,5,6,7]. 

The prevalence of malnutrition in these patients is estimated to be between 20 and 45%, depending on the series and the different methods used [5,6,7], and is often underdiagnosed. The cost of malnutrition in patients with COPD includes an increase in the use of resources, such as a greater number of hospitalizations, longer hospital stays, an increase in emergency department visits, as well as higher medical, pharmacy, and home oxygen therapy expenses. This ultimately translates into a higher expenditure per patient and year, as demonstrated by numerous studies [8,9,10].

Nutritional care in this pathology is of vital importance in the overall results of the treatment, but even more so in the aspects related to the patient’s quality of life throughout their entire disease process [11,12,13,14,15,16,17,18]. However, in most of the clinical practice guidelines (CPG) or reviews of more consolidated organizations [19,20,21,22,23,24,25], although they are updated year after year, the nutritional and dietary contents are scarce and do not provide a response tailored to the nutritional needs of these patients.

The objective of this review, therefore, is to respond to relevant questions that arise regarding the nutritional management of adult patients with COPD in terms of Morphofunctional Nutritional Assessment and intervention in routine clinical practice. These recommendations are specifically aimed at doctors, nurses, and dieticians involved in the care of these patients.

## 2. Methodology

The Andalusian Group for Nutrition Reflection and Investigation (GARIN) recommendations have been developed by a group of physicians specializing in endocrinology and nutrition with recognized expertise in clinical nutrition. Initially, the members of GARIN proposed a series of relevant issues in the clinical practice and nutritional management of patients with COPD. From these, the questions based on patient characteristics, Nutritional Screening and Morphofuntional Assessment, type of intervention, control, and outcome (PICO) were formulated to address the most individualized nutritional therapy according to the clinical characteristics of the patients and their level of risk or complexity. Finally, 24 recommendations were made in response to the PICO questions: 2(A), 6(B), 2(0), and 14(GPP). Of these,17 recommendations reached a consensus above 90% (indicating a strong consensus), and 7 obtained a consensus between ≥75–90%.

**Figure 1:**
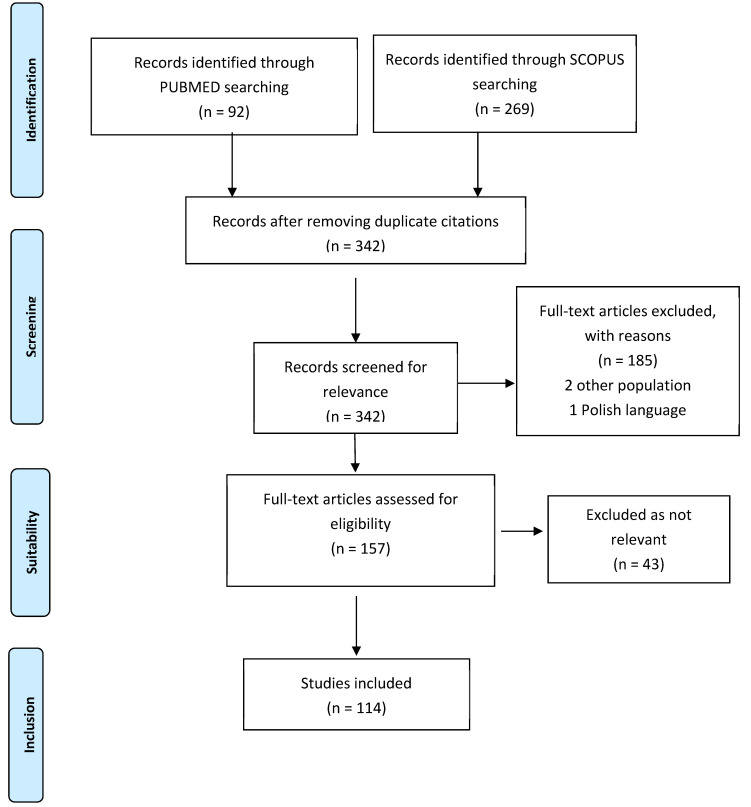
Flowchart following the PRISMA methodology.

To answer the PICO questions, a literature search was conducted in PUBMED and SCOPUS, filtered by systematic reviews, review articles, meta-analyses, and randomized clinical trials (RCTs) from the last 10 years, limited to adult subjects and published in English or Spanish. The keywords were ‘COPD’ or ‘chronic obstructive pulmonary disease’ and ‘nutrition’. A total of 361 results were obtained, and after eliminating duplicates, articles that did not meet the search criteria, and non-relevant articles, 114 articles were included. Figure 1 specifies the process according to the PRISMA (Preferred Reporting Items for Systematic Reviews and Meta-Analyses) methodology [26]. The critical appraisal of each article was carried out following the SIGN (Scottish Intercollegiate Guidelines Network) methodology [27], and articles were classified according to the checklist for each type of article (Table 1). The articles were reviewed by two independent reviewers (AJE and JMRR). In the case of doubt or discrepancy between reviewers, a third reviewer (MIRP) was consulted.

Finally, the draft article was circulated among all members until the final version was completed.

The wording of the recommendations reflects the degrees of evidence (Table 2) [28]; level A is indicated by the word ‘Recommend’, level B by the word ‘Suggest’, level 0 by the word ‘Advise’, and low quality of evidence by ‘It is currently not possible to make recommendations’. The ‘GPP’ recommendations are based on expert opinion because of a lack of studies; in these cases, the drafting was performed by group consensus. The first draft of the document was handed over to the GARIN members in July 2022, and once the document was revised, taking into account the comments and suggestions made by the group, it was sent for online voting in September 2022, applying a Likert scale of 1–5 (Table 3) [29]. The level of consensus for each recommendation was calculated by adding the total value resulting from the responses obtained, dividing it by the maximum value, and then multiplying by 100. Finally, the draft article was circulated among all members until the final version was completed.

## 3. Results and Discussion

Four questions were discarded because, in this review, sufficient evidence has not been found to make recommendations in this regard: two on energy requirements, one on the ideal proportion of macronutrients in the diet, and another on the specific use of nutritional supplements. Finally, 24 recommendations were made in response to the PICO questions: 2(A), 6(B), 2(0), and 14(GPP). Of these, 17 recommendations reached a consensus above 90% (indicating a strong consensus), and 7 obtained a consensus between ≥75–90%.

### 3.1. General

#### 3.1.1. Should Food Intake Be Assessed?

**Recommendation** **1.** *In patients with advanced COPD (GOLD 3 and 4: E), GARIN suggests routinely evaluating daily food intake at all levels of care, integrating dietary advice early that includes increasing the number of meals per day, with a focus on energy-rich foods and proteins, to help improve nutritional status and quality of life.*

Grade of recommendation GPP, (96%) Strong consensus.

Comment:

Decreased food intake is considered one of the main causes of malnutrition in COPD patients [16,30,31]. Malnourished patients have lower intakes and eat fewer meals per day compared with well-nourished patients [12,15,16]. Insufficient intake has been observed during periods of hospitalization [12], especially during exacerbations [32]. Food intake decreases in more advanced stages of COPD [30].

An energy and protein intake below 75% of requirements was considered a predictor of adverse events, and conversely, those patients with higher intakes had a trend of lower mortality risk [12].

Intake has been related to fat-free mass index (FFMI); in the Norden study [33], patients with low FFMI levels, regardless of severity, had a higher symptom burden affecting intake.

#### 3.1.2. When Should COPD Patients’ Weight Loss Be Assessed?

**Recommendation** **2.** *GARIN suggests assessing weight loss in all COPD patients from diagnosis and at each visit.*

Grade of recommendation B, (100%) Strong consensus.

Comment:

In COPD patients, progressive weight loss occurs even with adequate caloric intake, possibly conditioned by several circumstances, such as increased work of breathing, a state of systemic inflammation and sustained oxidative stress, acute exacerbations, and infectious complications [11,14,15,30,33]. 

In COPD, weight loss appears as a dynamic state that accelerates as the disease progresses [17] and is associated with increased morbidity and mortality [8,9,30,34,35,36]. Increased mortality has been reported in patients with weight loss of >5% in the previous 6 months [15], and weight gain of 2 kg has been found to be associated with improvements in muscle and respiratory strength [32]. 

In the JO study [8], COPD-related healthcare utilization and medical costs were higher among underweight patients than in the other groups.

#### 3.1.3. Is It Possible to Establish a BMI Range for COPD Patients?

**Recommendation** **3.** *GARIN suggests maintaining a body mass index (BMI) above 21 kg/m^2^ and below 30 kg/m^2^.*

Grade of recommendation B, (90%) Consensus.

Comment:

In many studies, BMI correlates positively with lung function [8,13,14,35,37,38] and negatively with exacerbations [38]. Patients with a BMI above 22 kg/m^2^ have been found to have a better prognosis and fewer exacerbations. Possible reasons include better nutrient intake, better nutritional and muscle status, and less inflammation. In some studies, overweight or obese patients even had better lung function, fewer exacerbations, and less inflammation than those with a BMI of less than 21 kg/m^2^ [8,38,39].

Low BMI has been associated with an increased risk of hospital admissions and long-term hospital stays [7] and is considered an independent risk factor for mortality in patients with COPD [7,8,30,34,35]. In the study by WU [38], overweight and obesity were associated with lower mortality rates compared with normal weight among smokers with COPD, but this association was not present among non-smokers with COPD. This could be explained by physiological differences between smoking-associated COPD and COPD in non-smokers [38]. Smoking is a risk factor for COPD and is associated with weight loss and other pathologies that increase the risk of mortality. It is a known central anorectic agent and also conditions the appearance of other cofactors that may influence weight loss [40]. Early implementation of smoking cessation measures can help slow the progression of the disease and reduce the risk of cardiovascular disease and mortality [41,42]. 

BMI is one of the determining factors of bone mineral density, and the effect of body weight seems to be influenced by both fat mass and lean mass. In addition to a low BMI, patients with COPD often present sarcopenia or low FFMI, and this association is mediated not only by mechanisms such as decreased skeletal load but also by hormonal and other specific factors such as systemic inflammation, vitamin D deficiency, and the use of oral or inhaled corticosteroids [43,44]. A relationship has been found between decreased bone mineral density and low BMI, with significantly high values as BMI and FFMI increase [45].

Despite this, the relationship between obesity and COPD is unclear, and it is difficult to establish whether obesity actually has a detrimental impact on COPD patients. The prevalence of obesity in COPD patients is estimated to range from 10% to 50% [46,47]. Some studies have reported worse respiratory symptoms, increased severe exacerbations, increased comorbidities, worse prognosis, greater restriction of daily activities, worse health-related quality of life, and more use of medical care in obese COPD patients [48,49].

It is clear that the exclusive assessment of BMI does not provide sufficient data to clarify the association of BMI with COPD. Assessing changes in body composition and muscle quality is mandatory. It is also essential for future research to explore the relevance of different phenotypes [15,50,51], taking into account different types of obesity, different symptoms (emphysematous, chronic bronchitis, or both), groups of smokers and non-smokers, and other relevant factors [51].

#### 3.1.4. Should Body Composition Be Assessed in the COPD Patient and When Should It Be Done?

**Recommendation** **4.** *GARIN suggests measuring muscle mass and function, both at diagnosis and at follow-up.*

Grade of recommendation B, (92%) Strong consensus.

Comment:

COPD is characterized by altered body composition, especially increased fat mass and decreased muscle mass [52]. Its influence on disease severity and prognosis has been described in numerous studies [12,14,35,53]. Because it is directly associated with lung function, its loss intensifies as disease severity increases. Morbidity, hospitalization rate, increased readmissions and hospital stay, and the need for ventilatory support are also increased in patients with significant muscle wasting [7,14,22,35,54,55].

Muscle mass is directly related to mortality; for some authors, FFMI is an independent predictor of mortality [7,33,56] and is suggested as a systemic marker of disease severity in COPD staging [35,56].

### 3.2. Nutritional Screening and Morphofuntional Assessment

#### 3.2.1. In Which Patients under Follow-Up for COPD Should Nutritional Screening Be Performed?

**Recommendation** **5.** *GARIN suggests performing nutritional screening in all patients diagnosed with COPD, regardless of the degree of severity and stage of the disease.*

Grade of recommendation GPP, (98%) Strong consensus.

Comment:

Extrapulmonary manifestations in COPD patients due to their chronic inflammatory state, including nutritional status, body composition, and changes in muscle mass, fat mass, and bone mineral density, have been shown to be determinants in the prognosis and severity of the disease, so nutritional screening is recommended for all patients diagnosed with COPD [11,15,35,57,58,59,60].

#### 3.2.2. What Nutritional Screening Tool Should Be Used?

**Recommendation** **6.** *GARIN advises the use of any validated screening tool, as there is no specific tool for detecting malnutrition in patients diagnosed with COPD.*

Grade of recommendation O, (98%) Strong consensus.

Comment:

It is essential to determine weight change as a percentage since increased mortality has been reported in patients with weight loss of >5% in the previous 6 months [15].

Validated nutritional screening tools most commonly used in studies of COPD patients include the Mini-Nutritional Assessment (MNA) [15,57,61,62] and MNA-SF [63], Geriatric Nutritional Risk Index (GNRI) in patients >65 years [64], Nutritional Risk Screening (NRS 2002) [35,60,65,66], Malnutrition Universal Screening Tool (MUST) [15], and Icelandic Screening Tool (ISS) [35].

The MNA questionnaire has been widely used in elderly patients for detecting those patients at risk of malnutrition even before weight changes occur, and has been found to be a predictive marker of mortality and hospital costs [62].

An association has been demonstrated with the number of exacerbations and the MNA-SF questionnaire, as well as with other pulmonary assessment parameters, including %FEV1 (forced expiratory volume in 1 s), %VC (vital capacity), %RV (residual volume), %DLCO (diffusing capacity for carbon monoxide) [63].

An association has been found between a low GNRI and a decreased 6 min walk distance (6MWD) value in COPD patients.

A decrease in weight and BMI has been described in those patients with a higher nutritional risk as measured by the NRS 2002 scale, together with worse lung function and lower exercise capacity [60], higher one-year mortality, and higher hospital readmissions (cut-off point < 3 points) [65].

Albumin level (within automated nutritional risk screening) is a good inflammatory marker associated with increased in-hospital mortality (cut-off point < 30.5 g/L) and increased risk of readmission (cut-off point < 30.1 g/L) [65].

#### 3.2.3. How to Establish the Degree of Malnutrition after Screening?

**Recommendation** **7.** *The patient’s degree of malnutrition should be previously established according to the latest ESPEN recommendations using the GLIM or SGA criteria (see Appendix A).*

Grade of recommendation A, (98%) Strong consensus.

Comment:

There are many studies in this field that use the Subjective Global Assessment (SGA) for the diagnosis of malnutrition [58,67,68,69,70,71,72]. 

GARIN members recommend nutritional diagnosis according to the latest ESPEN consensus guidelines [72], currently the GLIM or SGA criteria [11,35]. 

Based on the GLIM criteria, the diagnosis of malnutrition is reached when at least one phenotypic criterion (weight loss, age-related BMI, or Fat-Free Mass Index (FFMI)) coexists with another etiological criterion (reduction in intake/nutrient absorption or presence of inflammation/disease). The assessment of muscle mass becomes one of the phenotypic criteria in the diagnosis of malnutrition and the classification of its severity.

The SGA scale is made up of several items that provide a score to predict the patient’s nutritional status.

#### 3.2.4. Is It Necessary to Screen for Sarcopenia in These Patients?

**Recommendation** **8.** *GARIN suggests screening for sarcopenia in all patients diagnosed with COPD.*

Grade of recommendation GPP, (98%) Strong consensus.

Comment:

It has been shown that patients with low lung function, even healthy patients without COPD, are associated with decreased muscle mass [73].

The coexistence of COPD/asthma and sarcopenia in the elderly is common, and studies show a prevalence of around 15–25% [11,74]. These associations have been shown to further increase quality of life impairment and reduce the physical capacity for exercise [74].

Further, patients with pulmonary pathology and a higher degree of sarcopenia are associated with a higher percentage of osteoporosis, especially asthma–COPD overlap syndrome [75]. 

Patients with a low exercise capacity (6MWD < 350 m) and those with a decrease in physical activity (<7128 steps/day) are associated with an increase in exacerbations, a poorer quality of life, higher scores on depression scales, and decreased anthropometric and laboratory parameters related to disease prognosis and mortality [57].

Finally, a statistically significant relationship between the presence of sarcopenia and non-alcoholic fatty liver disease (NAFLD) (highly prevalent in COPD patients) has been determined after multivariate analysis, excluding other risk factors [76], as well as between sarcopenia and the presence of metabolic syndrome, mainly in men with a restrictive pulmonary pattern [77].

The members of GARIN, therefore, propose that, although no literature references have been found in the search, the use of new tools, such as the SARC-F, could be very useful in screening these patients for sarcopenia. 

#### 3.2.5. Which Tool Should We Use for the Diagnosis of Sarcopenia?

**Recommendation** **9.** *GARIN suggests making the diagnosis of sarcopenia according to the criteria included in the consensus algorithm established by the European Working Group on Sarcopenia in Older People (EWGSOP2) *[78]* (see Appendix B).*

Grade of recommendation GPP, (92%) Strong consensus.

Comment:

Among the different studies published, the most widely used and validated diagnostic method for determining sarcopenia in this group of patients is the EWGSOP2 algorithm criteria [11,74,79]. 

According to the EWGSOP2 guideline, the diagnosis of sarcopenia requires a decrease in muscle strength and mass associated with a lack of exercise resistance. Therefore, to establish it, it is necessary to assess muscle strength, mass, and functionality.

#### 3.2.6. How Should Muscle Function Be Assessed?

**Recommendation** **10.** *GARIN suggests the use of tests for the assessment of physical activity and exercise capacity, as they are useful for determining muscle function in COPD patients and offer reliable and comparable measurements over time and between individuals.*

Grade of recommendation GPP, (90%) Consensus.

Comment:

Within the COPD patient population, validated tests for the assessment of physical activity include 4 m gait speed (4MGS), 6MWD, and objective measurements of daily physical activity using pedometers or other devices [11,57,59,64]. Several studies have determined a low limit of less than 0.8 m/s for 4MGS [11].

Two-minute walking distance (2MWD) has also been assessed to detect frailer patients who show an increased risk of 18-month mortality, increased risk of dependency, higher rates of malnutrition, increased dyspnoea, and poorer quality of life test scores [80]. A clear correlation has been described between this and BMI, FEV1, the Medical Research Council dyspnoea score (mMRC), and 6MWD, with a cut-off point of ≤80 m.

Grip strength through dynamometry [11,37,74,79,81] has shown a positive correlation with muscle mass, lung function measured by spirometry, and the 6MWD test. A negative correlation between mMRC and morbidity and mortality has also been found [81].

Due to its wide use and ease of use, we especially recommend 6MWD and hand dynamometry.

#### 3.2.7. How to Assess Muscle Mass?

**Recommendation** **11.** *GARIN suggests assessing muscle mass using any of the following techniques depending on the availability and feasibility of each medical equipment.*

Grade of recommendation GPP, (82%) Consensus.

Depending on the techniques available, we can carry out a basic, intermediate, or advanced study of the patient’s muscle mass at both diagnosis and follow-up (Table 4).

Comment:

The gold standard for the assessment of bone mineral density and body composition is DEXA [15,57,59]. However, there are many studies in which bioelectric impedance analysis has been used, with a good correlation between fat-free mass and physical capacity, as well as a more severe staging of the disease, hospitalization for >7 days, and increased mortality at 6–9 months [11,15,35,79]. A decrease in fat-free mass of <15 kg/m^2^ in women and 16/17 kg/m^2^ in men or <10th percentile has been associated with increased mortality and morbidity [15,35,58].

Different studies have defined low muscle mass with cut-off points of skeletal muscle mass index (SMI) of ≤8.50 kg/m^2^ in men and ≤5.75 kg/m^2^ in women [11]. A significant reduction in phase angle has also been described, especially in patients with sarcopenia [11]. In addition to these two techniques, the assessment of muscle mass has been evaluated in various studies using CT, MRI, and muscle ultrasound [79]. A relationship between decreased fat-free mass/lean mass index and increased disease severity and increased mortality has been reported. Thus, it has been reported in cross-sectional and retrospective studies that the pectoralis major muscle, assessed through CT (in terms of area and density), is associated with various body composition, respiratory, and prognostic variables in COPD. However, further research is required to standardize its use in clinical practice [82,83,84]. 

Measurements of the anterior rectus femoris muscle (anteroposterior axis, area, etc.) at different levels (e.g., between the lateral epicondyle and the greater trochanter of the femur) have been performed by ultrasound, showing a significant correlation with the grip strength measured by dynamometry and the fat-free muscle mass by bioelectric impedance analysis [79].

In terms of anthropometry, the most commonly used indices are brachial circumference and calf circumference measured in cm (pathological < 31 cm) [74].

These techniques should always be performed by qualified professionals. In centers without experience in more complex techniques or in those where they are not available, anthropometric measurements such as circumferences and bioimpedance, if accessible, are recommended, in addition to collecting raw bioelectric data.

### 3.3. Nutritional Requirements

#### 3.3.1. How to Measure Energy Requirements in Adult COPD Patients?

**Recommendation** **12.** *In well-nourished and stable patients, GARIN advises the use of the following:*

*WHO or Harris–Benedict Equation × Activity Factor (AF);*

*Adjusted weight if BMI > 30.*

*In advanced-stage malnourished patients (GOLD 3 or GOLD 4: E):*

*Harris–Benedict Equation × FA × Disease Factor (DF) (1.3).*

Grade of recommendation GPP, (94%) Strong consensus.

Comment:

Several studies estimate an increase in basal energy expenditure (BEE) of 15–26% above the requirements of healthy individuals [59,85,86,87,88]. This increased metabolism has been associated with reduced food intake, weight loss, and muscle wasting and is considered an independent predictor of morbidity and mortality [85].

Although indirect calorimetry (IC) is the recommended method, when it is not available, predictive equations are a good option. 

Research that has compared different predictive equations with IC or the doubly labeled water (DWL) method has found that they overestimate or underestimate the REE. Thus, most of those studies recommend the Harris–Benedict and WHO equations as the ones that have best matched the values of the reference method used [59,86]. Rao [86] compared the resting energy expenditure (REE) measured by IC and the Harris–Benedict equation in mechanically ventilated patients. The IC-measured REE was approximately 45.0% higher (49.1% in males; 36.8% in females). In this study, the REE, according to the Harris–Benedict equation multiplied by 1.5 in men and by 1.4 in women, was close to the values obtained by IC. 

GARIN members reaffirm their interest in deepening the morphofunctional assessment of COPD patients and the possibility that, in the future, predictive equations may incorporate body composition data to allow a better adjustment of energy intake in these patients’ diets [88,89,90].

#### 3.3.2. What Are the Protein Requirements of the COPD Patient?

**Recommendation** **13.** *GARIN suggests an intake of 1 g protein/kg body weight/day in stable patients and 1.2–1.5 g/kg for malnourished patients in advanced stages and during exacerbations.*

Grade of recommendation B, (96%) Strong consensus.

Comment:

There is very limited evidence on specific protein requirements in COPD patients. The study by Kao [85] described, using the isotope tracer technique, an increase in protein metabolism that correlated with REE in COPD patients. Protein catabolism was not significantly different between COPD subjects and controls. On the other hand, Jonker et al. [90] found that threshold and anabolic capacity are preserved in clinically and weight-stable COPD patients and, therefore, suggest that there is no disease-related anabolic resistance and/or increased protein requirements [90]. Results obtained from studies on protein metabolism in vivo are inconclusive, and other studies report an increase in both synthesis and catabolism, suggesting an overall increase in protein metabolism compared with healthy subjects, in which the lower leucine concentrations were associated with low FFM in the COPD group [91]. 

On the other hand, there is some evidence in the reviewed literature on the benefits achieved in terms of increased muscle mass and strength in those patients who consume more protein [14,92,93,94].

Although the current study has not found any specific recommendations for COPD patients, the members of GARIN, based on the literature reviewed, consider it important to advocate a higher protein intake in the diet of these patients.

#### 3.3.3. What Is the Ideal Macronutrient Ratio in This Patient Group?

*There is insufficient evidence to make recommendations.*
Comment:

There is limited evidence on the impact of macronutrient distribution on the clinical course of the disease, and there are insufficient metabolic studies to provide an estimate of the ideal percentage distribution of carbohydrates, lipids, and proteins in the COPD patient. A respiratory quotient (RQ) of <1.0 is desirable in these patients, as the patient will exhale less carbon dioxide. However, this review has not found studies that evaluate carbohydrate and fat oxidation separately in COPD patients, and this is critical to understanding the energy metabolism of these individuals. Information on the oxidation of nutritional substrates in this specific population is lacking. In the search conducted, few studies have addressed this issue [14,85,87,90,95].

### 3.4. Nutritional Management

#### 3.4.1. What Type of Diet Would Be Indicated?

**Recommendation** **14.** *The diet recommendations given by the members of GARIN are shown in Table 5*.

Grade of recommendation GPP, (88%) Consensus

Comment

In general terms, different published reviews recommend the intake of a varied, healthy, and balanced diet for COPD patients without reducing the caloric intake because of the risk of associated malnutrition, trying to divide the intake into several meals to increase the caloric intake [58,96].

A decrease in the risk of developing various pathologies, including COPD, has also been observed in patients with an adequate intake of fish and a low proportion of saturated fats [15,97].

On the other hand, multiple epidemiological studies point to the protective potential of fruit and vegetables due to their high content of antioxidant substances (vitamins A and E) as well as fiber, anti-inflammatory properties, slowing of glucose and starch absorption, decreased lipid oxidation, and increase in anti-inflammatory cytokines by the gut microbiota. Thus, an adequate intake of these to prevent the development of the disease is recommended [58,98,99].

A study of 21,148 patients from the Korean National Health and Nutrition Examination Survey (2007–2014) showed that patients with higher intakes of vitamin A, carotenoids, and vitamin C had significantly higher FEV1 than those with lower intakes, as well as a lower risk of COPD, regardless of smoking status [100].

Another study [101] conducted on this population in 2012, with 3283 adults ≥40 years of age, 512 of whom were diagnosed with COPD, described how those with low intakes of nutrients, including potassium, vitamin A, carotenes, retinol, and vitamin C were at significant risk of developing COPD. In the multivariate analysis, gender, older age, smoking, and low vitamin C intake were independent risk factors for developing COPD. 

In contrast, a systematic review has shown how a 50 g/week increase in processed red meat intake leads to an increase in COPD risk of 8% [102]. Increased inspiratory limitation has also been reported in patients with higher consumption of carbonated beverages and coffee and increased smoking rates [103]. 

#### 3.4.2. What Is the Role of Dietary Advice?

**Recommendation** **15.** *GARIN recommends basic dietary advice as it is essential for COPD patients and the basis for proper lifestyle habits, especially in relation to diet.*

Grade of recommendation GPP, (100%) Consensus.

Comment:

Several clinical trials have studied the effect of dietary advice in COPD patients, showing an increase in caloric and protein intake, with an increase in weight and an improvement in SGA scores [70]. An increase in inspiratory strength and quality of life questionnaire scores has also been reported [73]. Several of these studies have also shown an improvement in clinical variables such as 6MWD or grip strength [104] and a significant reduction in the percentage of smokers, as well as better adherence to a Mediterranean diet and an increase in caloric intake with structured meal programs [105,106].

#### 3.4.3. In Patients with COPD and Malnutrition, Is Nutritional Supplementation Associated with an Improvement in Morphofunctional Nutritional Parameters and Disease Progression?

**Recommendation** **16.** *GARIN suggests the use of oral nutritional supplements (ONSs) in malnourished COPD patients to improve nutritional status and disease course (Table 6* *).*

Grade of recommendation B, (96%) Strong consensus.

*Comment—Weight and lean mass increase:*

The scientific evidence in this area has evolved over time. While more recent authors have reported increases in weight and lean mass, as well as improvements in exercise tolerance, earlier studies did not support these findings. 

Ferreira’s 2012 Cochrane Review [107] includes a meta-analysis of 17 RCTs with little evidence in favor of those patients receiving nutritional supplements in terms of improvement in weight, lean mass, respiratory muscle strength, and 6MWD, particularly striking in malnourished patients [37].

However, more recent studies show that the use of ONSs is associated with increased intake, improved anthropometric measures, and increased muscle strength [58,67,68,69,92,108,109,110,111,112]. 

A significant improvement in anthropometric measures (fat-free mass, brachial circumference, tricipital fold), 6MWD, ISWT, respiratory muscle strength (maximum inspiratory and expiratory pressure), and grip strength measured by dynamometry is described in the aforementioned studies [15,67,69,92,105,107,109,110,112,113,114,115].

*Comment—Potency of effect in physical exercise:*

Most studies, especially those focused on the assessment of fat-free mass, associate nutritional supplementation with regular physical exercise, so it is known that the association of the two enhances their effect [15,92,108,111,112,116].

*Comment—Improved muscle and lung function:*

Meta-analyses using different methods have been published, showing a significant association with daily calorie intake, grip strength, and quadriceps muscle strength [11,15,107].

*Comment—Improved quality of life:*

Improvements have been observed in the Chronic Respiratory Disease Questionnaire (CRQ) (which measures the presence of dyspnoea, fatigue, emotion, and mastery) and quality of life as measured by the St George’s Respiratory Questionnaire in undernourished patients with COPD, as well as anxiety and depression scores by HADS and the EQ-5D, in patients with nutritional supplementation [67,69,92,107,108,109,110,112,113,114,115].

*Comment—Improvement in laboratory parameters:*

A decrease in blood pressure and triglycerides and an increase in c-HDL have been reported with formulas enriched in omega-3 fatty acids, vitamin D, and high-biological-value proteins [92].

An improvement in laboratory parameters such as vitamin D, eicosapentaenoic acid (EPA) and docosahexaenoic acid (DHA), and inflammatory markers such as highly sensitive C-reactive protein, interleukin-6, interleukin-8, and tumor necrosis factor-alpha, has also been reported [67,112].

*Comment—Decreased mortality:*

Clinical trials with high protein supplements, some of them enriched in hydroxy methyl butyrate (HMB), have also shown decreased 90-day mortality rates, with a number needed to treat (NNT) of 20.3 [68,93]. A sub-analysis of the EFFORT study showed that these results were consistent in patients with lower respiratory tract infections [66].

#### 3.4.4. Is There a Specific ONS for This Group of Patients?

*There is insufficient evidence to make any recommendations about specific supplements in this group of patients.*
Comment:

Several supplements have been analyzed that have shown a significant benefit in the variables studied, most of them high-calorie and high-protein supplements enriched with certain micronutrients (omega-3, vitamin D, leucine, HMB) (Table 7).

Enteral nutrition formulas developed with a higher fat content (50–55%) have not shown a clear benefit in hospitalized COPD patients or those on mechanical ventilation [58].

#### 3.4.5. What Is the Role of Enteral and Parenteral Nutrition in Exacerbation Episodes in COPD Patients?

**Recommendation** **17.** *GARIN advises covering the necessary calorie intake in patients admitted for decompensated COPD, using the most physiological route possible at an early stage, as long as this provides adequate coverage of their requirements.*

Grade of recommendation GPP, (98%) Strong consensus.

Comment:

Intake in mechanically ventilated patients is decreased, mainly in those with oral nutrition, longer mechanical ventilation (MV), and higher BMI at admission [117].

In this review, we found only one non-systematic review study analyzing the role of nutritional support during exacerbations. Bordeje Laguna [58] recommends that in patients admitted to intensive care with the need for prolonged MV (>8 days) and high nutritional risk, receiving enteral nutrition and, failing that, total parenteral nutrition is associated with greater survival at 6 months and better recovery 3 months after discharge from the ICU. Likewise, patients with good nutritional coverage are more likely to be discharged home rather than to intermediate rehabilitation centers [58].

On the other hand, it has been determined that ventral decubitus does not contraindicate the use of enteral nutrition and is not associated with an increased risk of gastrointestinal complications or aspiration pneumonia [58].

A published clinical trial in 50 hospitalized patients showed that EPA administration at a dose of 1 g/day was not associated with significant benefits in terms of muscle mass preservation or hospital stay [118].

There is insufficient evidence to make specific recommendations on the use of glutamine, branched-chain amino acids, vitamins, or antioxidants in this group of acutely decompensated patients. 

#### 3.4.6. Is There Sufficient Evidence to Recommend Any Form of Micronutrient or Trace Element Supplementation?

**Recommendation** **18.** *Grade of recommendation according to GARIN’s position on supplementation with micronutrients and trace elements (Table 8* *).*

Grade of recommendation GPP, (90%) Consensus.

*Comment—HMB and essential amino acids:*

Supplements rich in leucine, essential amino acids, HMB, and creatine need further study in critically ill patients. 

Supplementation with HMB seems to provide the strongest evidence in this respect, with an improvement in nitrogen balance in patients with high catabolism. Administration of essential amino acids has been shown to improve body composition and nutritional status in other pathologies associated with increased muscle catabolism [119].

Studies conducted in patients with moderate/severe COPD [120] have shown that supplementation with a high proportion of leucine-enriched essential amino acids is associated with increased protein anabolism [32]. Increased physical capacity, fat-free mass, muscle strength, SaO_2_, serum albumin, and quality of life scales, as well as a decrease in cognitive impairment progression, have also been observed in patients with severe COPD who do not meet the criteria for participation in pulmonary rehabilitation programs [121].

*Comment—Omega-3, Vit D and leucine:*

There are numerous published interventions with a very heterogeneous methodology, including supplements enriched in carbohydrates and fatty acids, essential amino acids, whey protein rich in BCAAs, creatine, and polyunsaturated fatty acids (PUFAs) (natural ligands of peroxisome proliferator-activated receptors). Initial studies with fat-enriched supplements do not appear to show a significant benefit; however, more recent studies using carbohydrate and PUFA-enriched formulations appear to show improved physical training in selected patients [15].

In a study of 86 patients with moderate inspiratory limitation, low lung diffusion capacity, adequate protein intake, and decreased levels of vitamin D and DHA, the administration of a supplement enriched in omega-3, vitamin D, and leucine for 4 months in association with physical exercise (in both groups) showed statistically significant differences in the increase in muscle mass, levels of Vit D, EPA, and DHA, and the number of steps achieved [111]. 

Some studies also show a reduction in exacerbations after vitamin D supplementation in patients with previous deficiency [58,99].

Vitamin D supplementation is, therefore, recommended in patients with deficiency (<20 ng/mL), with a clear benefit in the prevention of falls, especially in association with calcium. A dose of 800 IU with 1 g of calcium is recommended. Supplementation with higher doses requires further study [15,122,123].

The addition of leucine and carbohydrates to protein supplements, either alone or in combination, has not been shown to increase protein anabolism compared with high-protein supplementation alone [94,116].

*Comment—Antioxidant vitamins:*

Several studies show antioxidant vitamin deficiency rates of up to 81% [58,99,124]. 

The use of magnesium and vitamin C-enriched whey formulations [67] has shown a decrease in inflammatory markers as well as an increase in fat-free mass, grip strength, and quality of life tests in patients with moderate/severe COPD. 

In different systematic reviews, vitamin C supplementation has not demonstrated relevant clinical benefits in patients with pulmonary pathology [99]. However, more recent publications show contradictory results. 

According to a published study, the administration of a supplement enriched in α-tocopherol (vitamin E) (30 mg/day), vitamin C (180 mg/day), zinc gluconate (15 mg/day), and selenomethionine (50 μg/day), associated with a physical exercise program, did not show an increase in exercise endurance, although it was associated with an increase in muscle strength and an increase in serum total protein levels [124]. Vitamin E administration in smoking patients showed a reduction in markers related to prostaglandin production, such as urine 8-iso-PGF2α by 21%, not significant compared with in combination with selenium or selenium administration alone [125]. 

### 3.5. Physical Activity

#### 3.5.1. What Is the Best Strategy in the Rehabilitation of the COPD Patient, Associated with Nutritional Therapy?

**Recommendation** **19.** *GARIN recommends pulmonary rehabilitation, combining aerobic and strength training exercises.*

Grade of recommendation A, (100%) Strong consensus.

**Recommendation** **20.** *GARIN suggests including at least 6–12 weeks of continuous physical training.*

Grade of recommendation GPP, (94%) Strong consensus.

**Recommendation** **21.** *GARIN suggests the interval modality for patients with severe COPD.*

Grade of recommendation B, (93%) Strong consensus.

Comment:

Pulmonary rehabilitation has established itself as one of the key strategies in the management of COPD patients. Pulmonary rehabilitation programs include physical exercise as an essential component of rehabilitation, in addition to other interventions such as education, dietary advice, and psychological support. Studies have shown that a comprehensive and intensive pulmonary rehabilitation program achieves significant improvements in clinical (dyspnoea, fatigue), body composition, physical capacity (exercise tolerance, muscle strength), and quality of life parameters [37,56,104,115,126]. 

One of the hallmarks of COPD is the progressive decline in physical exercise capacity due to skeletal muscle loss and dysfunction, which is a predictor of morbidity and mortality independent of lung function impairment [15,32,56,60,104,113,126,127,128]. The COVID-19 pandemic further exacerbated these effects, significantly reducing physical activity levels and potentially worsening muscle loss and dysfunction [129].

Muscle training has been widely shown to be effective in improving exercise tolerance, muscle strength, dyspnoea, fatigue, and quality of life [37,126,130].

Studies are currently focusing on identifying the essential components that achieve the best results in the short and long term, including the type and exercise intensity, frequency and duration of sessions, location (center or home), face-to-face supervision or other innovative strategies (tele rehabilitation), individualized or in groups.

Aerobic or endurance training is the most widely used form of exercise, for which there is the strongest recommendation evidence. A modification of standard aerobic training is interval training, where periods of maximal exertion are regularly alternated with equal periods of rest or lower-intensity exercise. In this way, patients achieve high levels of exertion but with less dyspnoea and fatigue, providing benefits equivalent to those of classical aerobic training [37,126]. Studies consistently show that interval training is one of the best treatments for the most severe COPD patients [124,131].

Muscle strength training has great potential compared with aerobic exercise to increase muscle mass and strength and the advantage of less cardio–respiratory compromise [80,126,127,132]. 

Most training programs achieve the best physiological results through a combination of the two types of exercise [115,126].

There is no consistent evidence to define the duration of training programs. Although studies of very variable duration (between 4 and 16 weeks) have been proposed [113,124,126], most of them consider that substantial benefits can be achieved with a duration between 6 and 12 weeks and with a frequency of 3–4 sessions per week [37,115,126,127,128,133].

#### 3.5.2. Is It Possible to Make a Specific Recommendation on Physical Exercise?

**Recommendation** **22.** *GARIN r* *ecommends regular physical activity according to the WHO guidelines for adults with chronic diseases.*

Grade of recommendation GPP, (96%) Strong consensus.

**Recommendation** **23.** *GARIN advises incorporating behavioral change strategies to increase physical activity engagement in COPD patients.*

Grade of recommendation O, (86%) Consensus.

Comment:

People with COPD are less active than people without COPD. Most reduce their activity levels in the early stages of the disease, walk at a slower pace, and generally do not meet the physical activity criteria recommended by the WHO. Patients avoid activities that involve physical exertion and increased symptoms, thus perpetuating a vicious cycle in which lack of exercise further compromises the physical ability to participate in any activity [30,37,56,104,134,135,136]. Physical inactivity leads to higher rates of morbidity, increased risk of premature mortality and hospitalization, and decreased quality of life [137]. Therefore, physical activity is identified as a potentially modifiable target that could be related to the patient’s disease progression and quality of life [30,113,135,138,139].

Although strategies to increase physical activity have been proposed, some studies have found that these only lead to a 2% increase in the time spent in daily activity, and therefore, recommendations to decrease the time spent in sedentary activities are advocated [137]. 

The members of GARIN have found no evidence of specific recommendations for physical activity in daily life for COPD patients and, therefore, consider that it is advisable to promote activities following the WHO recommendations for the general population [140]. 

### 3.6. Quality of Life

#### What Are the Most Commonly Used Quality of Life Questionnaires?

**Recommendation** **24.** *GARIN suggests the use of the following quality of life, anxiety, and depression questionnaires in the follow-up of COPD patients.*

*For the assessment of anxiety and depression* [57,108]:

*HADS anxiety score;*

*HADS depression score.*

*For the evaluation of the quality of life of patients with COPD, GARIN suggests the COPD Assessment Test (CAT)* [141] *in clinical practice. The St George’s Respiratory Questionnaire in undernourished patients with COPD (SGRQ)* [15,47,70,107,121,142] *and the Health-related quality of life using a validated version of the EuroQol five-dimensional (EQ-5D) questionnaire* [57,75,108,143,144]*, such as EQ-5D-5L index value or EQ-5D-5L VAS although they have been validated, are longer and more complex.*

Grade of recommendation GPP, (90%) Consensus.

## 4. Limitations and Strengths

Limitations: The principal limitation of the study is that only eight recommendations have been made with a high quality of evidence because there are important gaps in methods for assessing nutritional status, the impact of muscles in the process, nutritional requirements, and the most appropriate interventions in terms of nutrition, specific nutrients, and physical activity. The opinion of people with COPD and their caregivers has not been taken into account, nor are there pulmonology specialists in the group. Lastly, the final document was not sent to an external group for validation.

Strengths: A systematic review has been carried out with a methodology following the PRISMA criteria and applying grading of the evidence according to the SIGN (Scottish Intercollegiate Guidelines Network) methodology. In addition, an approximation has been made to the degree of consensus among experts in the area. Questions have been addressed with an eminently practical orientation.

## 5. Conclusions

Malnutrition is common in COPD patients and is associated with major complications that contribute to the frailty, morbidity, and mortality of COPD patients.

Members of GARIN recommend a multidisciplinary approach to COPD patients, integrating early Morphofunctional Nutritional Assessment and treatment to reduce the occurrence of complications linked to malnutrition and, thus, contribute to the patient’s well-being and improved quality of life. In pre-COPD and PRISM clinical presentations, it is important to implement preventive measures that could prevent disease progression or lead to early diagnosis and nutritional treatment [15,51,145,146].

Due to the limitations of the classic parameters for assessing nutritional status, a new global vision of clinical nutrition is necessary, integrating different aspects of the morphofunctional evaluation of patients with COPD, taking into account the different metabolic phenotypes [15,50] and their association with other phenotypes (emphysematous, chronic bronchitis, or both), which allows establishing a more precise diagnosis of the nutritional situation and an individualized therapeutic plan [51], as concluded by recently published studies [147].

These recommendations and suggestions on the nutritional management of the COPD patient have applicability in daily clinical practice; however, there are many questions about energy and nutrient requirements, and there are many uncertainties about the ideal percentage distribution of carbohydrates, lipids, and proteins in COPD patients’ diets. There is no consistent evidence to define the duration of training programs and the best strategy to increase physical activity in daily life. Further studies are needed to increase the quality of the evidence and provide specific answers to many of these questions.

Randomized and preferably double-blind clinical trials evaluating the impact of nutritional therapy in different clinical situations in both inpatient and outpatient settings would be desirable. Studies comparing different enteral nutrition formulas in COPD patients should evaluate the efficacy and efficiency (cost-effectiveness) of these formulas on metabolic effects and morbidity/mortality in order to make evidence-based recommendations.

## Figures and Tables

**Table 1:** Levels of evidence (LE) [27].

1++	High-quality meta-analyses, systematic reviews of RCTs, or RCTs with a very low risk of bias
1+	Meta-analyses, systematic reviews of well-conducted RCTs, or RCTs with low risk of bias
1−	Meta-analyses, systematic reviews of well-conducted RCTs, or RCTs at high risk of bias
2++	High-quality systematic reviews of case-control studies; cohort or case-control studies with a low risk of confounding, bias, or chance and a high probability that the relationship is causal
2+	Well-conducted case-control or cohort studies with a low risk of confounding, bias, or chance and a moderate probability that the relationship is causal
2−	Case-control or cohort studies with a high risk of confounding, bias, or chance and a significant risk that the relationship is causal
3	Non-analytical studies, such as case reports and case series
4	Expert opinion

**Table 2:** Grades of recommendation [28].

A	At least one meta-analysis, systematic review, or RCT rated 1++ and directly applicable to the target population; or A body of evidence consisting primarily of studies rated 1+, directly applicable to the target population, and demonstrating the overall consistency of the results
B	A body of evidence that includes studies rated as 2++, directly applicable to the target population; or a body of evidence that includes studies rated as 2+, directly applicable to the target population and demonstrating overall consistency of results; or extrapolated evidence from studies rated 1++ or 1+
O	Level of evidence 3 or 4; or extrapolated evidence from studies rated 2++ or 2+
GPP	Good Practice Point/Expert Consensus: Recommended best practice based on the clinical experience of the expert panel

**Table 3:** Likert scale [29].

1	Strongly disagree
2	Disagree
3	Neither agree nor disagree
4	Agree
5	Strongly agree

**Table 4:** Muscle mass assessment techniques according to the degree of complexity and availability.

	Level of Study
Dual-energy X-ray absorptiometry (DEXA)	***
Magnetic resonance imaging (MRI)	**
Computed tomography (CT)	**
Muscle ultrasound	**
Bioelectric impedance analysis	**
Anthropometry	*

*** Advanced; ** Intermediate; * Basic.

**Table 5:** The diet recommended by GARIN.

In stable patients, a varied, healthy and balanced diet, such as the Mediterranean dietary pattern, as in the general population. Do not decrease caloric intake to achieve less work of breathing because of the risk of malnutrition associated with a BMI below 21 kg/m^2^. Eat fish 2–3 times a week. Split the intake into several meals (5–6 meals/day) in case of advanced COPD GOLD 3 or 4 and during exacerbations. Educate patients on healthy fat choices with a decrease in industrial saturated and trans fats. Eat a diet high in fruit and vegetables because of their high antioxidant and fiber content (five servings per day). Decrease intake of processed meats and carbonated beverages.

**Table 6:** The degree of recommendation according to GARIN’s position on each of the aspects under consideration.

Objectives to Be Taken into Account	Grade of Recommendation
Weight increase	***
Lean mass increase	***
Potency of effect in physical exercise	***
Improvement in muscle functionality	**
Improvement in lung function	**
Improvement in quality of life	**
Improvement of laboratory parameters	*
Reduction of post-hospitalisation mortality	*

*** Recommend; ** Suggest; * Advise.

**Table 7:** Studies analyzing oral supplementation in patients with COPD.

ONS (kcal/prot)	(*N*) and Time	Grade COPD	Comparator	Effects	References
Normo/Normo Omega-3/6 DHA FOS (Prebiotic fiber)	(99) 3 months	Stable outpatient Mild/moderate/severe	PCB	↑ weight ↑ FEV1 ↑ Grip strength ↑ 6MWT	Benito Martinez et al. [109]
High/High Leucine Omega-3 [111] PUFAs [108] Vitamin D	(81) 4 months [108,111] (45) 3 months [92]	Stable outpatient Moderate/severe +High-intensity exercise	PCB [108,111] Isocaloric formula [92]	↑ Vit D, EPA, DHA, Omega-3 ↑ Weight, ↑ FM ↑ FEV1 ↓ Depression Score without difference with isocaloric formula * [92]	Van de Bool et al. [111] Van Beers et al. [108] Calder et al. [92]
High/High HMB	(652) 3 months [68,69] (214) 3 months [93]	Hospital COPD > 65 years + other pathologies. Up to 90 days post-H	PCB P (comp form 48 kcal standard + Vit C) [68]	↓ 30–60–90 day mortality (NNT 20) ↑ SGA scale ↑ Weight ↑ Vit D ↑ Grip strength ↑ nutritional markers	Deutz et al. [68] Deutz et al. [93] (sub-analysis of [68]) Matheson et al. [69]
Normo/High Whey protein Omega-3 Vitamins A,C,E	(36) 3 months (12) 2 weeks	Stable outpatient Moderate/severe +Low-intensity exercise	PCB [112] Hydrolysed casein [113]	↓ Inflammatory markers ↑ 6MWT ↑ Weight, ↑ FM ↑ Quadriceps strength ↑ Inspiratory pressure max ↑ Emotional Function and Quality of Life score No difference between the two types of protein [113]	Sugawara et al. [112] Jonker et al. [116]
Low (0.45)/High Whey protein Magnesium + Vitamin C	(44) 2 months	Stable outpatient Moderate/severe	PCB	↑ FFM ↑ Vit C ↑ St. George’s Respiratory Questionnaire ↓ Inflammatory markers	Ahmadi et al. [67]

Abbreviations: ONS = oral nutritional supplement; N = number of patients included; PCB = placebo (understood as dietary advice); DHA = docosahexaenoic acid; 6MWT = 6 min walking test; EPA = eicosapentaenoic acid; FM = fat mass; FFM = fat-free mass. * milk-based comparator that contained no 25-hydroxy-vitamin D3, milk protein instead of pure whey protein, and sunflower oil in place of omega-3 polyunsaturated fatty acid (PUFA) containing fish oil. ↑ = Increase; ↓ = decrease.

**Table 8:** Grade of recommendation according to GARIN’s position on supplementation with micronutrients and trace elements.

	Grade of Recommendation
Monitor levels of ions, especially magnesium, calcium, phosphorus	***
Vit D if deficient (<20 ng/mL)	**
HMB and essential amino acids	*
Omega-3, Vit D and leucine	N
Antioxidant vitamins A, C and E, and selenium	N

*** Recommend; ** Suggest; * Advise; N: Not positioning.

**Figure A1:**
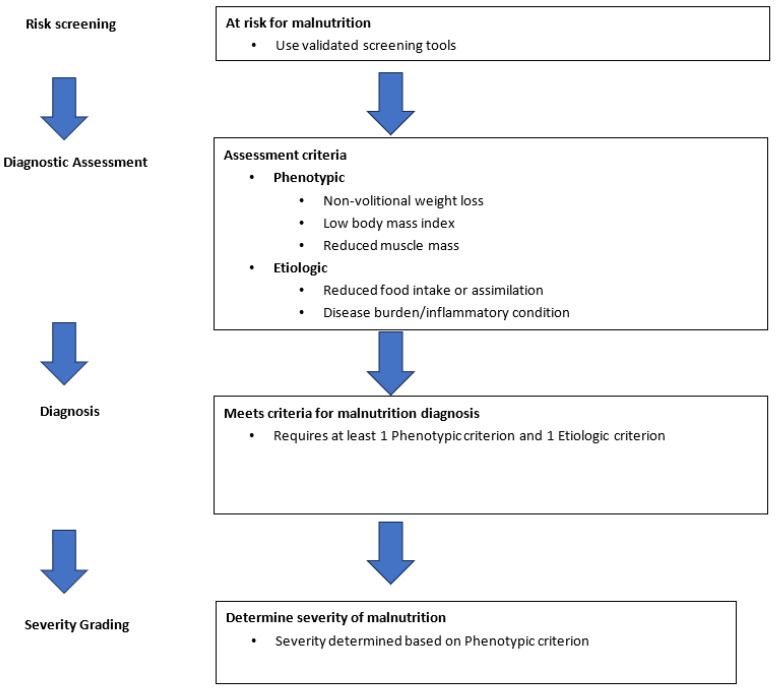
GLIM diagnostic scheme for screening, assessment, diagnosis and grading of malnutrition. Adapted from ref. [148].

**Figure A2:**
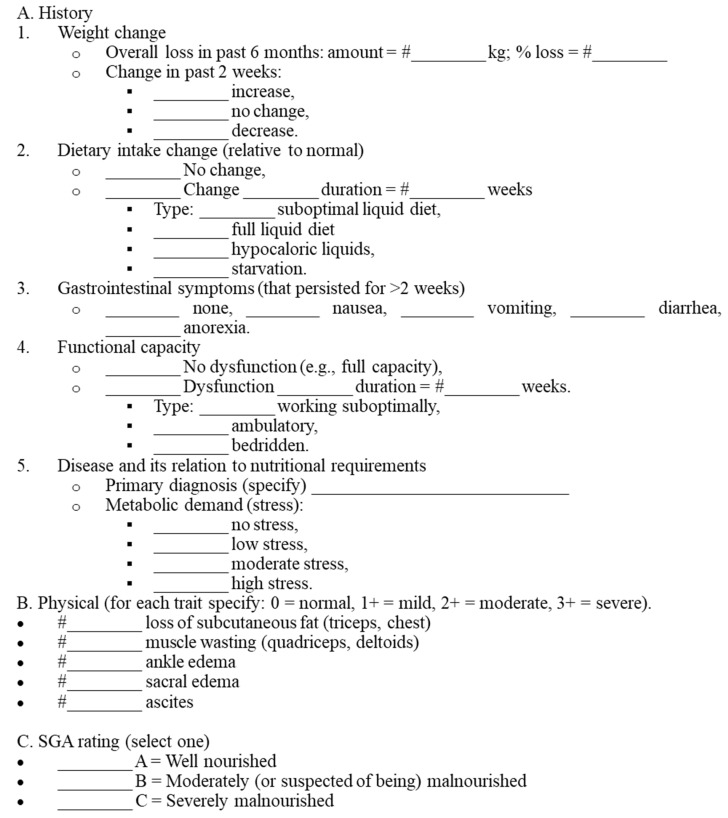
Items contained in the Subjective Global Assessment (SGA). Adapted from ref. [148].

**Figure A3:**
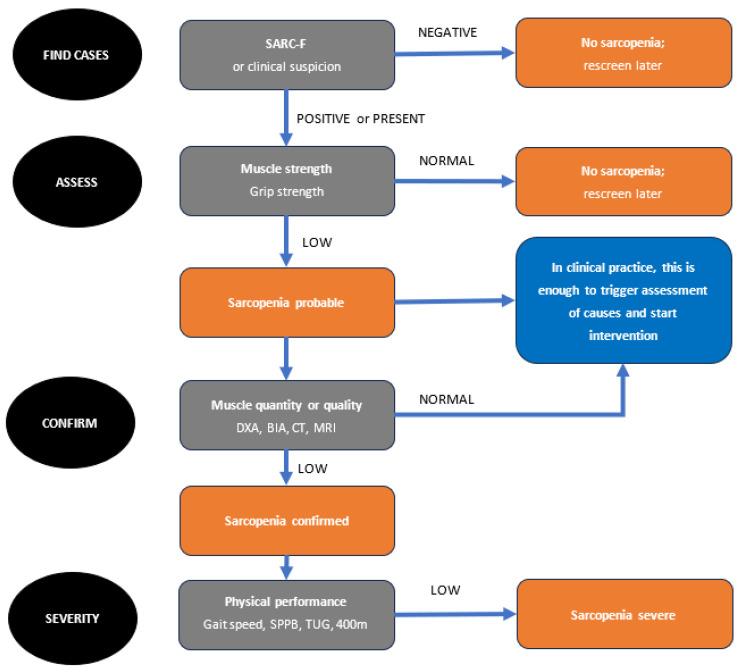
Algorythm for case-finding making a diagnosis and quantifying severity in practice. The steps of the pathway are represented as Find-Assess-Confirm-Severity or F-A-C-S “Consider other reason for low muscle strength (e.g., depression, stroke, balance disorders, peripheral vascular disorders)”. Abbrevations: DXA = Dual-energy X-ray absorptiomety; BIA = Bioelectrical impedance analysis; CT = computed tomography; MRI = magnetic resonance imaging; SPPB = short physical performance battery; TUG = timed-up-and-go test. Adapted from ref. [78].

## Appendix A. Glim Criteria and SGA

**Table A1:** GLIM tresholds for severity grading of malnutrition into Stage 1 (Moderate) and Stage 2 (Severe) malnutrition. Adapted from ref. [148].

	Phenotypic Criteria ^a^		
	Weight Loss (%)	Low Body Mass Index (kg/m^2^) ^b^	Reduced Muscle Mass ^c^
**Stage 1 (Moderate Malnutrition)**	5–10% within the past 6 mo,	<20 if < 70 yr, <22 if ≥70 yr	Mild to moderate deficit
(Requires 1 phenotypic criterion that meets this grade)	or 10–20% beyond 6 mo		(per validated assessment methods—see below)
**Stage 2 (Severe Malnutrition)**	>10% within the past 6 mo,	<18.5 if < 70 yr, <20 if ≥70 yr	Severe deficit
(Requires 1 phenotypic criterion that meets this grade)	or >20% beyond 6 mo		(per validated assessment methods—see below)
(a) Severity grading is based upon the noted phenotypic criteria while the etiologic criteria described in the text and Figure 1 are used to provide the context to guide intervention and anticipated outcomes. (b) Further research is needed to secure consensus reference BMI data for Asian populations in clinical settings. (c) For example appendicular lean mass index (ALMI, kg/m^2^) by dual-energy absorptiometry or corresponding standards using other body composition methods like bioelectrical impedance analysis (BIA), CT or MRI. When not available or by regional preference, physical examination or standard anthropometric measures like mid-arm muscle or calf circumferences may be used. Functional assessments like hand-grip strength may be used as a supportive measure.			

## Appendix B. EWGSOP2 and SARC-F

**Table A2:** SARC-F SCALE. Adapted from ref. [149].

Component	Question	Scoring
Strength	How much difficulty do you have in lifting and carrying 10 pounds?	None = 0, Some = 1, A lot or unable = 2
Assistance in walking	How much difficulty do you have walking across a room?	None = 0, Some = 1, A lot, use aids, or unable = 2
Rise from a chair	How much difficulty do you have transferring from a chair or bed?	None = 0, Some = 1, A lot or unable without help = 2
Climb stairs	How much difficulty do you have climbing a flight of 10 stairs?	None = 0, Some = 1, A lot or unable = 2
Falls	How many times have you fallen in the past year?	None = 0, 1–3 falls = 1, ≥4 falls = 2
